# Review: neuroestrogen regulation of socio-sexual behavior of males

**DOI:** 10.3389/fnins.2014.00323

**Published:** 2014-10-13

**Authors:** Takayoshi Ubuka, Kazuyoshi Tsutsui

**Affiliations:** Department of Biology and Center for Medical Life Science, Waseda UniversityShinjuku, Tokyo, Japan

**Keywords:** neuroestrogen, aromatase, socio-sexual behavior, aggressive behavior, sexual behavior, gonadotropin-inhibitory hormone, glutamate, dopamine

## Abstract

It is thought that estrogen (neuroestrogen) synthesized by the action of aromatase in the brain from testosterone activates male socio-sexual behaviors, such as aggression and sexual behavior in birds. We recently found that gonadotropin-inhibitory hormone (GnIH), a hypothalamic neuropeptide, inhibits socio-sexual behaviors of male quail by directly activating aromatase and increasing neuroestrogen synthesis in the preoptic area (POA). The POA is thought to be the most critical site of aromatization and neuroestrogen action for the regulation of socio-sexual behavior of male birds. We concluded that GnIH inhibits socio-sexual behaviors of male quail by increasing neuroestrogen concentration beyond its optimal concentration in the brain for expression of socio-sexual behavior. On the other hand, it has been reported that dopamine and glutamate, which stimulate male socio-sexual behavior in birds and mammals, inhibit the activity of aromatase in the POA. Multiple studies also report that the activity of aromatase or neuroestrogen is negatively correlated with changes in male socio-sexual behavior in fish, birds, and mammals including humans. Here, we review previous studies that investigated the role of neuroestrogen in the regulation of male socio-sexual behavior and reconsider the hypothesis that neuroestrogen activates male socio-sexual behavior in vertebrates. It is considered that basal concentration of neuroestrogen is required for the maintenance of male socio-sexual behavior but higher concentration of neuroestrogen may inhibit male socio-sexual behavior.

## Introduction

Originally it was considered that males display male-typical behavior because they are exposed to androgen secreted by the testis, whereas females display female-typical behavior because they are exposed to female sex hormones secreted by the ovary, such as 17β-estradiol (E2) and progesterone (Reviewed in Beach, 1948; Balthazart et al., 2004). However, it was later discovered that estrogen is able to activate male-typical behavior in castrated male rats (Beach, 1942). As the male-typical behavior activated by androgen can be blocked by concomitant antiestrogen treatment (Beyer and Vidal, 1971) and because the anterior hypothalamus can synthesize estrogens (neuroestrogen) from androgens by aromatization (Naftolin et al., 1972, 1975), it was hypothesized that central actions of androgen in males require its aromatization into neuroestrogen in the brain (aromatization hypothesis; Yahr, 1979). It was confirmed that aromatizable androgens such as testosterone or androstenedione can activate male sexual behavior in castrates, but non-aromatizable androgen such as 5α-dihydrotestosterone (5α-DHT) has little or no effect in mammals (McDonald et al., 1970; Whalen and Luttge, 1971) and birds (Adkins, 1977; Adkins et al., 1980; Harding et al., 1983). Aromatase inhibitors, such as Fadrozole (FAD) and Vorozole, inhibited or blocked the effect of testosterone on male sexual behavior in mammals (Christensen and Clemens, 1975; Beyer et al., 1976; Morali et al., 1977; Roselli et al., 2003) and birds (Adkins et al., 1980; Walters and Harding, 1988; Balthazart et al., 1990; Schlinger and Callard, 1990; Soma et al., 1999, 2000). It was further shown that male copulatory behavior was severely impaired in the aromatase knockout (ArKO) mouse (Fisher et al., 1998; Honda et al., 1998; Toda et al., 2001b; Matsumoto et al., 2003). Testosterone administration to castrated ArKO mice did not rescue copulatory behavior, but combined treatment with E2 and dihydrotestosterone (DHT) almost completely rescued it (Bakker et al., 2004).

It is widely accepted that the actions of neuroestrogen in the brain are mediated by estrogen receptor α (ERα) and β (ERβ) that belong to the nuclear receptor superfamily, leading to transcriptional regulation of the target genes (Tsai and O'Malley, 1994). It has been shown that E2 can increase cAMP in the uterus of ovariectomized mice within 15 s (Szego and Davis, 1967) suggesting non-genomic actions of E2. As genomic actions of estrogens take hours for changes in protein expression to occur, non-genomic actions of estrogens are defined as rapid effects occurring within seconds to minutes that are generally initiated at the plasma membrane, resulting in the activation of signal transduction pathways, such as kinase activation or calcium flux (Vasudevan and Pfaff, 2008). It is also becoming clear that that the activity of aromatase itself is rapidly regulated by non-genomic mechanism, such as direct phosphorylation of the enzyme (Balthazart et al., 2003; Roselli et al., 2009; Cornil et al., 2012). These results suggest that some factors in the brain may rapidly regulate socio-sexual behaviors of males by controlling the activity of aromatase and neuroestrogen synthesis. Candidates include glutamate and dopamine as they have been reported to rapidly inhibit the activity of aromatase in the brain (Balthazart et al., 2001a,b, 2002, 2006).

Gonadotropin-inhibitory hormone (GnIH) is a hypothalamic neuropeptide that inhibits gonadotropin secretion from the pituitary in birds and mammals (Tsutsui et al., 2000; Kriegsfeld et al., 2006; Ubuka et al., 2006, 2009a, 2012a; for reviews, see Tsutsui, 2009; Tsutsui et al., 2009, 2010; Ubuka and Bentley, 2011; Tsutsui and Ubuka, 2013; Ubuka et al., 2013c). GnIH expression is regulated by daily rhythm or melatonin (Ubuka et al., 2005), stress or glucocorticoid (Kirby et al., 2009; Son et al., 2014), and social environment (Tobari et al., 2014). In birds GnIH is synthesized in the paraventricular nucleus (PVN) in neurons that project to the median eminence (Tsutsui et al., 2000; Ubuka et al., 2003; Ukena et al., 2003). Abundant GnIH-immunoreactive (ir) fibers are observed in the preoptic area (POA) and the periaqueductal central gray (PAG) (Ubuka et al., 2008), where mRNA of the cognate G protein-coupled receptor (GPR147) for GnIH is expressed (Yin et al., 2005; Ubuka et al., 2008). As the POA and PAG are brain areas that regulate socio-sexual behaviors such as aggression and sexual behavior (Absil et al., 2001; Cornil et al., 2012), GnIH released in these brain areas may modify socio-sexual behaviors (Ubuka et al., 2012b, 2013b,c). The medial preoptic area (MPOA) is thought to play an important role in the regulation of male sexual behavior, because damage to the MPOA impairs sexual behavior (Klaric and Hendricks, 1986; Liu et al., 1997; Paredes et al., 1998), whereas MPOA stimulation enhances behavior (Malsbury, 1971; Paredes et al., 1990; Rodríguez-Manzo et al., 2000). The major efferent projections from the MPOA are to hypothalamic, midbrain, and brain stem nuclei that regulate autonomic or somatomotor patterns and motivational states (Simerly and Swanson, 1988).

Male socio-sexual behavior of birds is androgen dependent because it is reduced by castration and restored by androgen treatment (Selinger and Bermant, 1967; Mills et al., 1997), however there is no correlation between the order of aggressiveness and peripheral testosterone concentration (Tsutsui and Ishii, 1981). It is thought that the complete expression of testosterone action requires its aromatization into E2 in the brain, because socio-sexual behaviors of reproductively inactive male birds are only activated by aromatizable androgen, such as testosterone and androstenedione, or E2, but not by non-aromatizable androgen, such as DHT. Indeed the co-administration of aromatase inhibitors blocks testosterone-induced aggression in male quail (Tsutsui and Ishii, 1981; Schlinger and Callard, 1990). Ubuka et al. (2014) hypothesized that GnIH may inhibit socio-sexual behaviors of male quail by regulating aromatase activity and neuroestrogen synthesis in the brain. Their findings suggest that GnIH inhibits socio-sexual behaviors of male quail by directly activating aromatase and increasing neuroestrogen concentration in the POA beyond its optimal concentration (Ubuka et al., 2014; Ubuka and Tsutsui, 2014).

Here we review previous studies that investigated the role of neuroestrogen in the regulation of male socio-sexual behaviors and reconsider the hypothesis that neuroestrogen activates male socio-sexual behaviors in vertebrates. It is proposed that basal concentration of neuroestrogen is required for the maintenance of male socio-sexual behaviors but higher concentration of neuroestrogen may inhibit male socio-sexual behaviors in vertebrates.

## Molecular mechanisms regulating the activity of aromatase and male socio-sexual behavior in birds and mammals

### Action of dopamine in mammals

Dopamine facilitates sexual behavior in a number of species including humans (Bitran and Hull, 1987; Melis and Argiolas, 1995). Male estrogen receptor α knock-out (ERαKO) mice do not exhibit male-typical sexual behaviors (Wersinger et al., 1997), but treating ERαKO males with apomorphine, a non-selective dopamine agonist which activates both D1-like and D2-like dopamine receptors, stimulated male-typical copulatory behavior (Wersinger and Rissman, 2000a). Dopamine is thought to enhance sensorimotor integration by removing tonic inhibition (Chevalier and Deniau, 1990). Dopamine is not thought to directly elicit behavior, but it is thought to allow hormonally primed output pathways to have easier access to sexually relevant stimuli (Hull et al., 1999).

Three major integrative systems, the nigrostriatal system, the mesolimbic system, and the medial preoptic system, are thought to control sexual motivation and genital and somatomotor responses in male rats. Sensory input from a receptive female and/or copulation elicits the release of dopamine in each of these three integrative systems (Hull et al., 1999). The nigrostriatal system enhances both the readiness to respond to stimuli and motor integration; the mesolimbic system is critical for appetitive behavior and reinforcement, a motivational aspects of behavior but not only sexual motivation; and the medial preoptic system may focus the male's motivation on sexually relevant stimuli, coordinate the genital reflexes necessary for erection and ejaculation, and enhance species-typical motor patterns of copulation (Hull et al., 1999).

Dopaminergic input to the MPOA arises from the periventricular system, including cell bodies in the medial portion of the MPOA and the anterior portion of the incertohypothalamic tract (Simerly et al., 1986). The MPOA is one site where dopamine may promote sexual behavior, because dopamine agonists microinjected into the MPOA facilitate sexual behavior (Hull et al., 1986; Markowski et al., 1994), whereas microinjections of a dopamine antagonist impair copulation, genital reflexes, and sexual motivation to some extent (Pehek et al., 1988; Warner et al., 1991). Extracellular dopamine increases in the MPOA of male rats during precopulatory exposure to an estrous female and during copulation (Hull et al., 1995) and it is thought that both dopamine receptor subtypes (D1 and D2 receptors) are involved in the initiation and rate of copulatory behavior (Blackburn et al., 1992).

### Action of dopamine in birds

Kleitz-Nelson et al. (2010a) developed an *in vivo* microdialysis system to measure dopamine release in the MPOA of quail. Males failed to copulate with a female in the absence of a pre-copulatory rise in dopamine. In contrast, males that showed a substantial increase in MPOA dopamine during pre-copulatory interactions copulated with females. As there was no difference in dopamine during periods when the quail were copulating as compared to when the female was present but the males were not copulating, dopamine action in the MPOA was thought to be linked to sexual motivation rather than copulatory behavior (Kleitz-Nelson et al., 2010a). Kleitz-Nelson et al. (2010b) investigated the role of D1 and D2 receptors on male sexual behavior by examining how intracerebroventricular (i.c.v.) injections and microinjections of D1 and D2 agonists and antagonists into the MPOA influenced sexual behavior in male quail. I.c.v. injections of D1 or D2 agonists and antagonists indicated that D1 receptors facilitated consummatory male sexual behavior, whereas D2 receptors inhibited both appetitive and consummatory behavior.

**Figure 1:**
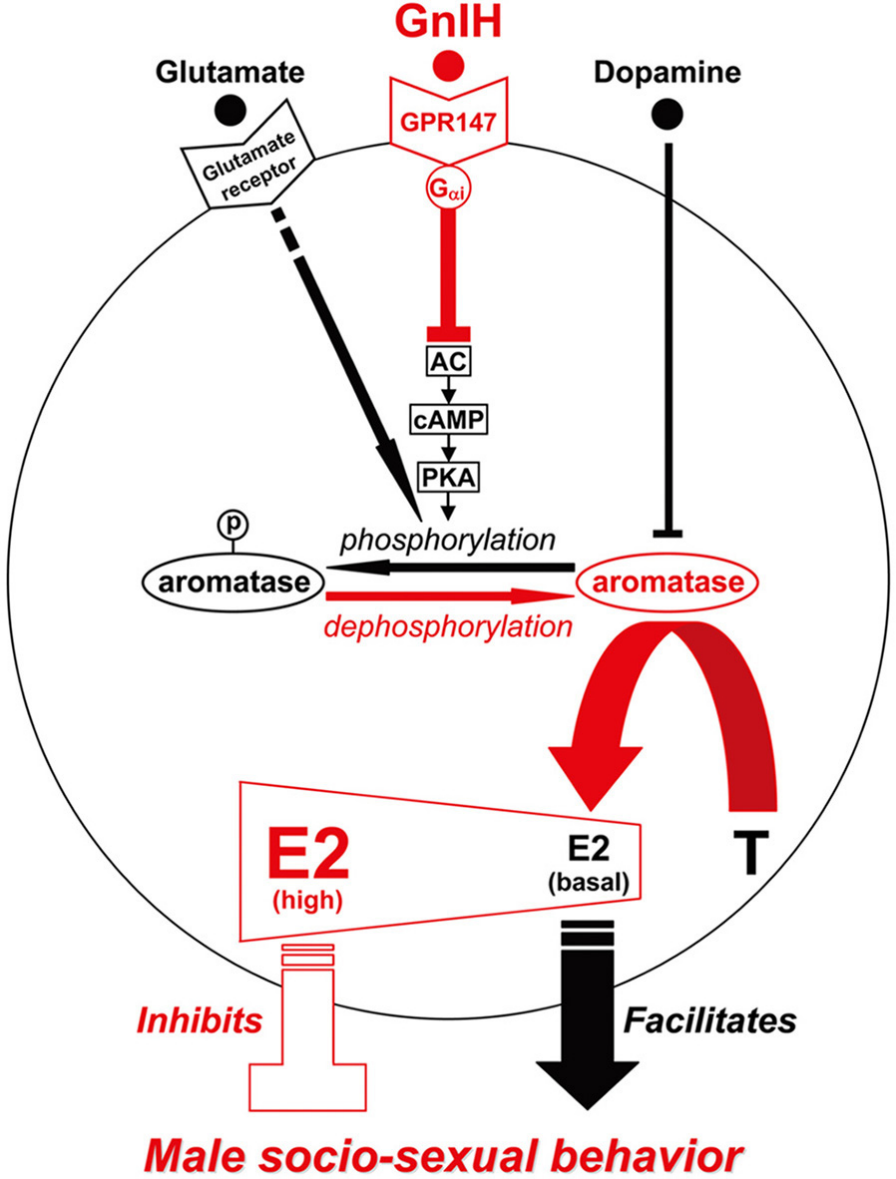
**Model of the intracellular mechanism of GnIH and its receptor (GPR147), glutamate and its receptor, dopamine that may control male socio-sexual behavior by regulating the activity of aromatase and neuroestrogen synthesis in the brain**. GPR147 is expressed on aromatase immunoreactive cells in the brain. GPR147 is coupled to G_α*i*_ protein that inhibits the activity of adenylate cyclase (AC) and decreases cAMP production and the activity of protein kinase A (PKA). Inhibition of AC/cAMP/PKA pathway may thus decrease phosphorylated aromatase and increase dephosphorylated aromatase. 17β-estradiol (E2) synthesized from androgen such as testosterone (T) by aromatase in the brain especially in the preoptic area (POA) regulates male aggression. It has been previously demonstrated that aromatase activity is rapidly down-regulated by phosphorylation, and this down-regulation is blocked by kinase inhibitors. The administration of GnIH activates aromatase by decreasing phosphorylated aromatase, and stimulates neuroestrogen synthesis in the brain. Aromatase activity and estrogen concentration in the brain especially in the POA are low in the morning when the birds are active, but aromatase activity and E2 concentration gradually increased until the evening when the birds became inactive. E2 release in the POA also increased in the evening. Finally, centrally administered E2 at higher doses in the morning inhibited aggressive behavior. These results suggest that GnIH inhibits aggressive behavior by directly activating aromatase and increasing neuroestrogen synthesis in the brain beyond its optimum concentration for the expression of aggressive behavior. Glutamate was shown to decrease the activity of aromatase by phosphorylation, and dopamine may act as an alternative substrate for aromatase to compete with testosterone and prevent its transformation into estrogens. Glutamate and dopamine may thus facilitate male socio-sexual behavior by decreasing the activity of aromatase and maintaining the optimum concentration of neuroestrogen for the expression of male socio-sexual behavior.

Immunohistochemical studies have demonstrated that there are dense networks of tyrosine hydroxylase (TH)-ir fibers in brain areas that contain aromatase-ir neurons, such as the sexually dimorphic MPOA or the bed nucleus striae terminalis (BNST) in quail. Double-labeling has confirmed that aromatase-ir cells are in close association with TH-ir fibers in quail (Balthazart et al., 1998). Therefore, the possible existence of a direct modulation of aromatase activity by dopamine and/or norepinephrine was systematically investigated by *in vitro* incubations of quail hypothalamic homogenates (Balthazart et al., 2002). Aromatase activity was quantified by the production of tritiated water from [1β −^3^H] androstenedione (Baillien and Balthazart, 1997). Norepinephrine had no or very limited effects on aromatase activity. In contrast, dopamine and several D1 and/or D2 receptor agonists [apomorphine (for both D1/D2), SKF-38393 (for D1) and RU-24213 (for D2)] depressed aromatase activity. As the inhibitory effect of the agonists was not antagonized by the D1 antagonist SCH-23390 or the D2 antagonist spiperone, the inhibitory effects of dopamine or dopaminergic compounds were thought not to be mediated through binding to dopamine receptors. Instead dopamine was thought to act as an alternative substrate for aromatase to compete with testosterone and prevent its transformation into neuroestrogens (Balthazart et al., 2002). Accordingly, dopamine should be transported into the aromatase cells in the MPOA by dopamine transporter or internalization of dopamine receptors to inhibit the activity of aromatase existing in the cytosol (Figure 1).

### Regulation of aromatase activity by phosphorylation

Several consensus sites of phosphorylation are present in aromatase sequences in mammals and birds (Corbin et al., 1988; Harada, 1988; Harada et al., 1992; McPhaul et al., 1988; Means et al., 1989; Shen et al., 1994), so it was hypothesized that phosphorylation may regulate the aromatase activity (Balthazart et al., 2001a,b). Balthazart et al. (2001a) demonstrated that aromatase activity in quail hypothalamic homogenates was rapidly down-regulated by adding Ca^2+^, Mg^2+^, ATP, conditions that enhance protein phosphorylation, and this inhibition of aromatase activity was blocked by kinase inhibitors (Balthazart et al., 2001b).

### Action of glutamate in birds

Balthazart et al. (2006) further showed that aromatase activity in quail hypothalamic explants was decreased within minutes by glutamate agonists (kainate, AMPA or NMDA), possibly by enhancing intracellular Ca^2+^ concentration and phosphorylation of aromatase. Cornil et al. (2000) visualized the distribution of the major ionotropic glutamate receptors in the quail brain by using primary antibodies raised against rat glutamate receptor 1 and receptors 2–3 (GluR1, GluR2/3: AMPA subtype), glutamate receptors 5–7 (GluR5–7: kainate subtype), and NMDA receptors (NMDAR1). The four types of receptors were broadly distributed in the brain. In particular immunoreactive cells are identified within the major aromatase cell groups located in the MPOA, ventromedial hypothalamus, nucleus striae terminalis, and nucleus taeniae. Dense populations of glutamate receptor-ir cells were also present with a receptor subtype-specific distribution in broad areas of the telencephalon (Cornil et al., 2000).

### Action of glutamate in mammals

Dominguez et al. (2006) measured glutamate in microdialysate samples from the MPOA before, during, and after copulation by male rats. There was a slight rise in extracellular glutamate when the female was presented, a significant increase during periods of mounting and intromitting, and a very large increase in samples collected during ejaculation with a precipitous fall in the first post ejaculatory sample. Dominguez et al. (2006) also administered a mixture of glutamate uptake inhibitors into the MPOA before and during mating by retromicrodialysis. The mixture increased extracellular glutamate and increased the number of ejaculations in the 40 min test, decreased ejaculation latency, and decreased the post ejaculatory latency to resume copulation. These results strongly suggest that MPOA glutamate is a major facilitator of copulation and the post ejaculatory fall in glutamate regulates the post ejaculatory interval (Dominguez et al., 2006). The results obtained in several species suggest that glutamate facilitates male sexual behavior by decreasing the activity of aromatase by phosphorylation in the MPOA (Figure 1).

### Action of GnIH in birds

Ubuka et al. (2014) first measured daily changes in the frequency of aggressive behavior of male quail and tested the effect of i.c.v. administration of GnIH on the frequency of aggressive behavior of male quail in the morning when its natural expression is high. I.c.v. administration of GnIH rapidly inhibited the number of male-typical aggressive behaviors of quail.

As previous studies suggested that full expression of testosterone action in the brain requires its aromatization in birds (Yahr, 1979; Tsutsui and Ishii, 1981; Balthazart and Surlemont, 1990; Schlinger and Callard, 1990; Panzica et al., 1996; Balthazart et al., 2009, 2011), Ubuka et al. (2014) hypothesized that GnIH may inhibit aggressive behavior of male quail by regulating neuroestrogen synthesis in the brain. Abundant GnIH-ir neuronal fibers and aromatase-ir cells were observed in the POA, BNST, mediobasal hypothalamus (MBH), and PAG, where aromatase mRNA is distinctively expressed in the quail brain (Voigt et al., 2007). Merged image of GnIH-ir neuronal fibers and aromatase-ir cells showed close appositions of GnIH-ir neuronal fibers in the vicinity of aromatase-ir cells in these brain areas (Ubuka et al., 2014). *In situ* hybridization for GPR147 mRNA combined with aromatase immunohistochemistry in the POA further showed that almost all aromatase-ir cells observed in the POA expressed GPR147 mRNA.

The effect of GnIH administration on aromatase activity and E2 synthesis in the POA *in vitro* and *in vivo* was examined by Ubuka et al. (2014). GnIH increased the activity of aromatase and E2 in an organ cultured brain block including the POA in a dose dependent manner. Ubuka et al. (2014) have also shown that the administration of a GnIH receptor antagonist RF9 (Simonin et al., 2006; Pineda et al., 2010) or an aromatase inhibitor FAD (Steele et al., 1987; Wade et al., 1994) canceled the stimulatory action of GnIH on E2 synthesis. Together these results indicate that GnIH increases neuroestrogen concentration by increasing the activity of aromatase after binding to GPR147 expressed on aromatase cells in the POA (Ubuka et al., 2014).

It was previously demonstrated that aromatase activity in hypothalamic homogenates of male quail is rapidly down-regulated by phosphorylation, and this inhibition is blocked by kinase inhibitors (Balthazart et al., 2001a,b, 2003, 2006; Charlier et al., 2011a). In order to investigate if GnIH activates aromatase by dephosphorylation of phosphorylated aromatase, Ubuka et al. (2014) measured phosphorylated aromatase by the Phos-Tag SDS PAGE method (Kinoshita et al., 2006) in the brain block including the POA of birds that were centrally administered with GnIH or vehicle in the morning. I.c.v. administration of GnIH reduced phosphorylated aromatase in the POA 30 min after administration (Ubuka et al., 2014).

Aromatase activity is not only controlled in the long term (hours to days) by transcription of the aromatase gene by steroids, but also in the short term (minutes) by phosphorylation by neurotransmitters, such as glutamate (Balthazart et al., 2006). GnIH was shown to be the first neuropeptide that can stimulate aromatase activity in the medium term (minutes to hours) (Ubuka et al., 2014). GnIH receptor GPR147 has been shown to couple predominantly through the G_α*i*_ protein to inhibit cAMP production in mammals (Hinuma et al., 2000; Ubuka et al., 2009b, 2012c, 2013c; Son et al., 2012). Son et al. (2012) investigated the cell signaling process of GPR147 using Lβ T2 cells, a mouse gonadotrope cell line, and it was shown that GnIH inhibits gonadotropin-releasing hormone (GnRH) induced gonadotropin subunit gene transcriptions by inhibiting adenylate cyclase (AC)/cAMP/PKA dependent ERK phosphorylation. As mentioned above, the action of GnIH on E2 synthesis in the POA was prevented by concomitant administration of RF9, a potent GPR147 antagonist, or FAD, an aromatase inhibitor (Ubuka et al., 2014). Ubuka et al. (2014) further demonstrated that i.c.v. administration of GnIH reduces phosphorylated aromatase in the POA. Previous studies have shown that aromatase activity is inhibited by phosphorylation in hypothalamic and ovarian homogenates of quail (Balthazart et al., 2001a,b, 2003) and in various cell lines transfected with human aromatase (Charlier et al., 2011a). Accordingly, it is highly possible that GnIH stimulates neuroestrogen synthesis in the POA by activating aromatase through dephosphorylation after binding to GPR147 expressed on aromatase cells (Figure 1).

## Environmental or social factors that modulate aromatase activity in male birds

### Effect of daily rhythm

When sexually active male quail are paired in a relatively small cage they fight using sequential aggressive actions. They often threaten the opponent by stretching the neck and walking around (strutting), approach and chase, peck the opponent (pecking), grab the back of the opponent's head or neck with their beak (grabbing), attempt to mount the opponent (mounting), mounting the opponent and lowering their cloaca close to the opponent's cloaca (cloacal contact (CC)-like actions). The frequency of these actions represents the activity of aggressive or sexual behavior of male quail (Selinger and Bermant, 1967; Tsutsui and Ishii, 1981; Schlinger and Callard, 1990; Mills et al., 1997).

Ubuka et al. (2014) quantified strutting, pecking, grabbing, mounting, and CC-like actions in 5 min during the light hours around zietgeiber time (ZT) 3, 6, 9, and 12 h. All male quail used in the experiment were kept under long day photoperiods (16 h light, 8 h dark) to keep them sexually active. The frequency of strutting, pecking, and grabbing actions was significantly higher in the morning (ZT 3 h) and decreased in the afternoon (ZT 9 h) and the evening (ZT 12 h). The frequency of mounting and CC-like actions was also high in the morning and tended to decrease until the evening.

Aromatase activity was assessed by measuring the conversion of [^3^H]androstenedione to [^3^H]E2 using brain homogenates or organ cultured quail brain blocks (Ubuka et al., 2014). Aromatase activity in the brain block including the POA or BSTM was low in the morning (ZT 3 h) and increased in the evening (ZT 12 h). The change in aromatase activity in the other brain blocks showed similar trends. E2 content and release in the POA was also low in the morning (ZT 3 h) and increased in the evening (ZT 12 h) possibly by the action of activated aromatase by dephosphorylation. Ubuka et al. (2014) also measured daily changes in E2 and testosterone concentrations in the serum, because changes in aromatase activity or E2 concentration in the brain may have reflected changes in E2 or testosterone concentration in the circulation. However, there was no daily change in E2 and testosterone concentrations in the serum.

### Effect of social interaction

Cornil et al. (2005) measured aromatase activity in hypothalamic/preoptic area (HPOA) homogenates of male quail following visual access to or copulation with a female. Sexual interactions resulted in a decrease in aromatase activity that reached its maximum after 5 min (Cornil et al., 2005). The time course of the effect of copulation on aromatase activity was also measured specifically in the different populations in the brain expressing high levels of aromatase activity (Schumacher and Balthazart, 1987) of male quail that experienced varying durations of visual exposure to or copulation with a female by the Palkovits punch method (de Bournonville et al., 2013). Sexual interactions resulted in a rapid inhibition of aromatase activity in specific brain regions including the MPOA and the tuberal hypothalamus (de Bournonville et al., 2013). The rapid decrease in neuroestrogen concentration in the MPOA may be important during the motivational phase of the behavior to trigger physiological events essential to activate mate search and thus copulation.

### Effect of stress

Balthazart et al. (2009) showed that exposing male quail to acute restraint stress for 15 min or injecting corticosterone 30 min before brain collection results in a significant increase in aromatase activity in HPOA homogenates. Dickens et al. (2011) investigated the effects of acute stress on aromatase activity in both sexes by measuring enzyme activity in all aromatase-expressing brain nuclei before, during, and after 30 min of acute restraint stress. Acute stress rapidly increased aromatase activity in the male MPOA in 5 min. This elevated activity persisted as long as the stressor was present and returned to control levels within 30 min after stress cessation (Dickens et al., 2011). These results suggest that stress rapidly increases aromatase activity in the brain of birds.

## Aromatase activity, neuroestrogen concentration and socio-sexual behavior of male vertebrates

### Studies in fish

Huffman et al. (2013) tested the role of aromatase in mediating aggression and reproductive behavior of male *Astatotilapia burtoni*, an African cichlid fish that display plasticity in social behavior. They found that subordinate males have higher aromatase expression than dominant males in the magnocellular and gigantocellular regions of the POA that regulate social behavior. Intraperitoneal injections into dominant male fish with FAD decreased aggressive, but not reproductive behavior. Indeed FAD treated males had increased aromatase expression in the gigantocellular portion of the POA (Huffman et al., 2013). These results suggest aromatase expression in the POA is negatively correlated with dominance or aggression in male *A. burtoni*.

Black et al. (2005) investigated the effect of social environmental change on aggressive behavior and brain aromatase activity in a sex-changing fish, *Lythrypnus dalli*. Male removal from a socially stable group results in rapid increases in aggression in the dominant female, which will later become male. These dominant females, and recently sex-changed individuals, had lower brain aromatase activity compared with control females and the established males had the lowest brain aromatase activity. Within hours of male removal, dominant females' aggressive behavior was inversely related to brain aromatase activity (Black et al., 2005). These results suggest that high E2 concentration in the brain caused by higher aromatase activity may inhibit aggressive behavior so that E2 concentration and aromatase activity should be reduced to increase aggressiveness and dominance within the social group.

Lord et al. (2009) tested the effects of testosterone, E2, and FAD on approach responses toward females in male goldfish (*Carassius auratus*). Injections of testosterone stimulated approach responses toward the visual cues of females 30–45 min later. E2 produced the same effect 30–45 min and even 10–25 min after administration and treatment with FAD blocked the exogenous effect of testosterone. The authors suggest that the testosterone surge induced by sexual stimuli may rapidly prime males to mate by increasing sensitivity within visual pathways that guide approach responses toward females and/or by increasing the motivation to approach potential mates. These actions occur within traditional limbic circuits, and the aromatization of testosterone maybe important for the male approach response toward females (Lord et al., 2009).

### Studies in birds

Historically studies in birds that have reported the involvement of aromatase in male sexual behavior and the stimulatory effect of E2 on male sexual behavior have used castrated male quail (Adkins, 1977) or reproductively inactivated male quail by photoperiodic manipulation (Adkins et al., 1980). Single doses of various steroids were administered peripherally to reproductively inactive birds for days or weeks to compare their effects (Adkins, 1977; Adkins et al., 1980; Tsutsui and Ishii, 1981; Wada, 1982; Schlinger and Callard, 1990). These studies are likely to have shown the genomic effects of E2 and other sex steroids on the brain that facilitated socio-sexual behaviors, which were attenuated by castration or photoperiodic manipulation.

Silverin et al. (2004) investigated the relationships among territorial aggression and brain aromatase activity in pied flycatcher, *Ficedula hypoleuca*, at the peak of the reproductive season. Aggressive behavior was measured during a simulated territorial intrusion in unpaired males holding primary territories. A significant correlation was observed between number of attacks/min displayed during the simulated territorial intrusion and aromatase activity in the anterior diencephalon but not in the posterior diencephalon and telencephalon (Silverin et al., 2004). These results suggest that aromatase activity in the anterior diencephalon is important for territorial aggression. Charlier et al. (2011b) exposed wild male white-crowned sparrows in the late breeding season to simulated territorial intrusion (STI) (song playback and live decoy) for 30 min. Although STI significantly increased aggressive behavior aromatase activity was not affected in the brain regions collected using the Palkovits punch technique. STI did not affect circulating levels of E2, but rapidly reduced E2 concentrations in the hippocampus, ventromedial nucleus of the hypothalamus and bed nucleus of the stria terminalis (Charlier et al., 2011b).

Many species also defend territories in the non-breeding season, when circulating testosterone levels are low. Castration of the western male song sparrow *Melospiza melodia morphna* had no effect on aggression in the non-breeding season, suggesting that autumnal territoriality is independent of gonadal hormones. Soma et al. (2000) treated wild, free-living non-breeding male song sparrows with FAD using micro-osmotic pumps. FAD greatly reduced aggressive behavior, and the effects of FAD were rescued by E2 replacement. These data indicate that E2 regulates male aggression despite low circulating levels of sex steroids or despite castration (Soma et al., 2000). Studies in diverse avian and mammalian species suggested that adrenal dehydroepiandrosterone (DHEA), an androgen precursor and prohormone, is important for aggressive behavior when gonadal testosterone is low and circulating DHEA can be converted into active sex steroids within the brain (Soma et al., 2014).

To investigate the physiological role of GnIH in the stimulation of E2 synthesis in the brain, Ubuka et al. (2014) analyzed the effects of i.c.v. administration of GnIH on E2 concentration in the brain and aggressiveness (peck frequency against the standard bird) of individual birds. I.c.v. administration of GnIH increased E2 concentration in the brain blocks including the POA or PAG, 30 min after administration. This was associated with a significant decrease in the frequency of pecking in the morning (ZT 2–4 h). As i.c.v. administration of GnIH stimulated E2 synthesis in the brain and inhibited the frequency of pecking actions, it was hypothesized that the high concentration of E2 in the brain may inhibit aggressive behavior. To test this hypothesis Ubuka et al. (2014) centrally administered various doses of E2 and measured five stereotypic actions of aggressive behavior in the morning (ZT 2–6 h). I.c.v. administration of E2 at 1 ng increased the frequency of CC-like action compared with vehicle administered birds. However, i.c.v. administrations of E2 at 10 ng, 100 ng, 1 μ g, and 10 μ g inhibited the frequency of pecking, grabbing, mounting, and CC-like actions compared with vehicle or 1 ng E2 administered birds (Ubuka et al., 2014). These results suggest that high concentrations of neuroestrogen inhibit socio-sexual behaviors of male quail although basal concentration of neuroestrogen facilitates socio-sexual behaviors (Ubuka and Tsutsui, 2014).

### Studies in rodents

Compaan et al. (1994) measured the brain aromatase activity in the POA, amygdaloid nuclei (Am), ventromedial hypothalamus (VMH), and parietal cortex (CTX) from two strains of adult male house mice, which were genetically selected for territorial aggression, based upon their attack latencies (short attack latency: SAL; long attack latency: LAL). Non aggressive LAL males had higher aromatase activity in the POA compared to aggressive SAL animals. The aromatase activity levels in both the VMH and Am did not differ significantly between strains. Aromatase activity was higher in POA than VMH in nonaggressive LAL males, whereas aromatase activity was higher in VMH than POA in aggressive SAL males. In both selection lines, the Am exhibited the highest levels of aromatase activity, as compared to the other investigated areas (Compaan et al., 1994).

Toda et al. (2001a,b) generated ArKO mice by targeting disruption of the CYP19 (aromatase) gene. They observed that ArKO males exhibited a complete loss of aggressive behavior in a resident-intruder paradigm. The behavior of ArKO males was partially reinstated when the mice received supplements of E2 soon after birth until the day of testing, but it was not restored when the supplementation was started at 7 days after birth (Toda et al., 2001a,b). These results suggest that neuroestrogen is required to construct neuronal infrastructure for aggressive behavior after birth and to maintain it in adult male mice.

Harada et al. (2009) also generated ArKO mice, which showed undetectable estrogen and enhanced androgen levels in blood. These ArKO mice exhibited enhanced appetite and displayed disorders in sexual motivation, sexual partnership preference, sexual performance, aggressive behavior, parental behavior, infanticide behavior and exploratory (anxiety) behavior. By introducing a transgene of human aromatase, controlled by the minimal promoter region, into the ArKO mouse they showed near recovery from behavioral disorders. This transgenic mouse line (ArKO/hArom) have a POA, hypothalamus and amygdala that are exposed to neuroestrogen only in the perinatal period, and then to enhanced androgens but no neuroestrogen exposure in adulthood, These results suggest that neuroestrogen acting in specific brain regions are important to organize sex-specific neural networks during the perinatal period (Harada et al., 2009).

### Studies in non human primates

Phoenix (1974) studied the sexual and sex-related behavior of adult male rhesus monkeys castrated 3 years earlier in pair tests with receptive females. The performance before, and during, daily treatment with 1 mg/kg dihydrotestosterone propionate (DHTP), a non-aromatizable androgen, was compared. It was shown that DHTP effectively rendered the performance level of the castrates comparable to that of the intact controls (Phoenix, 1974). This result suggests that aromatization of androgen is not required for male sexual behavior in this monkey species.

Zumpe et al. (1996) tested the effect of medroxyprogesterone acetate (MPA) (that reduces androgen uptake by brain), FAD, and E2 on the sexual motivation and behavior of castrated and testosterone treated male cynomolgus monkeys, *Macaca fascicularis*. Sexual motivation reflected in mounting attempts and mounting attempt latencies was diminished by E2 treatment in males receiving both MPA and FAD, but ejaculatory activity was unchanged (Zumpe et al., 1996). These results suggest that although testosterone and basal concentration of neuroestrogen is required for sexual motivation of males, higher concentration of neuroestrogen may inhibit sexual motivation reflected in mounting attempts.

### Studies in humans

Gooren (1985) reported that administration of tamoxifen, estrogen receptor antagonist, or testolactone, an aromatase inhibitor, had no effect on male human sexual function. Replacement of testosterone substitution therapy of agonadal men by DHT, non-aromatizable androgen, was not associated with any change of sexual functioning. Administration of DHT to eugonadal men led to a transient increase in nocturnal sexual dreams, erections and irritability. It was concluded that aromatization of testosterone is not required and that DHT maintains sexual functions in the adult male with an established sex life (Gooren, 1985).

Kyomen et al. (1999) performed a randomized, double-blind, placebo-controlled clinical trial to investigate the efficacy and safety of short-term estrogen therapy in decreasing aggressive behavior in elderly patients with moderate-to-severe dementia. They found that estrogen therapy was associated with lower total aggression scores and with decreased frequency of physical aggression over the 4-week trial and no adverse effects of estrogen were observed during the course of the study (Kyomen et al., 1999).

Orengo et al. (2002) investigated if testosterone and estrogen levels correlate with aggression in older men with dementia. Plasma total and free testosterone and estrogen levels and scores for behavioral disturbances, in particular aggression, were measured in elderly males who had a diagnosis of dementia. They found that free testosterone levels showed significant positive correlations with measures of aggression, but plasma estrogen levels showed significant negative correlations with measures of aggression (Orengo et al., 2002).

## Conclusion and possible mechanism

In this review we give an account of studies that have investigated the role of neuroestrogen or estrogen on socio-sexual behavior of males. Many correlational studies in fish, birds, and mammals suggest that male aggression or sexual behavior and aromatase activities in the brain are negatively correlated. Basal activity of aromatase appears to be required for male socio-sexual behaviors especially during development when neuronal infrastructure for male socio-sexual behavior is constructed or organized in the brain. As administration of aromatase inhibitor such as FAD decreases socio-sexual behaviors of adult males in many animals, aromatase and neuroestrogen seem to be also important for the maintenance of neuronal infrastructure for male socio-sexual behavior in adulthood. However, neuroestrogen may not be important for the maintenance of male socio-sexual behavior in some monkeys and humans. We speculate that this may be because of the relative roles that a developed cerebrum plays in the socio-sexual behavior of primates. Higher concentrations of neuroestrogen or estrogen may inhibit aggressive behavior in adulthood that was experimentally shown in male quail and elderly human males with dementia.

Although dopamine and glutamate stimulate male socio-sexual behaviors in birds and mammals, it was shown that they inhibit aromatase activity in the POA that is thought to be the most critical site of aromatization and neuroestrogen action for the regulation of male socio-sexual behaviors. These results further suggest that higher concentration of neuroestrogen especially in the POA may inhibit male socio-sexual behavior. Dopamine may act as an alternative substrate for aromatase to compete with testosterone and prevent its transformation into neuroestrogen. Accordingly, dopamine may facilitate male socio-sexual behavior by decreasing aromatase activity in the cytosol after it enters the cell through dopamine transporter or receptor internalization. Glutamate was shown to decrease the activity of aromatase by phosphorylation of aromatase, whereas GnIH increases the activity of aromatase by its dephosphorylation. The effects of glutamate and GnIH on phosphorylation or dephosphorylation of aromatase are likely to be achieved by cell signaling processes triggered after binding to their receptors. Even if dopamine, glutamate, and GnIH can rapidly change the activity of aromatase and neuroestrogen concentration in the POA, we consider that neuroestrogen in the POA may not directly regulate the movement of the body to perform socio-sexual behaviors because synthesized neuroestrogen could not be degraded in milliseconds after constrictions and relaxations of related muscles, instead different concentrations of neuroestrogen is likely to facilitate or inhibit the action of neurotransmitters and neuromodulators, including dopamine, glutamate, and GnIH, which are released according to social or natural, favorable or unfavorable environment.

The key question arising from the above hypothesis is what is the possible mechanism of neuroestrogen action according to its concentration from facilitation to inhibition on male socio-sexual behavior? It was shown that ERαKO male mice display decreased aggression toward intruders in resident-intruder tests (Ogawa et al., 1997). In contrast ERβ KO male mice are more aggressive than wild type mice in resident-intruder tests (Ogawa et al., 1999). There are also studies showing different roles of ER subtypes on the behavior. ERα was shown to be essential for female-directed chemo-investigatory behavior of males (Wersinger and Rissman, 2000b) and ERβ was shown to regulate anxiety behavior (Choleris et al., 2003; Imwalle et al., 2005; Lund et al., 2005). It may be possible that neuroestrogen regulates different ER subtypes depending on its concentration in the brain. Further studies including detailed analyses of the localization of aromatase and ER subtypes and the time-course of their activations are required to answer this question.

### Conflict of interest statement

The authors declare that the research was conducted in the absence of any commercial or financial relationships that could be construed as a potential conflict of interest.
